# Hospitalization for medical conditions in adolescent and young adult cancer survivors: a nationwide cohort study using the Netherlands Cancer Registry

**DOI:** 10.1007/s11764-026-01996-5

**Published:** 2026-03-07

**Authors:** Annemiek S. Lieffering, Chantal R. M. Lammens, Joyce Wilbers, Jourik A. Gietema, Winette T. A. van der Graaf, Olga Husson

**Affiliations:** 1 Department of Public Health, Erasmus University Medical Center, Rotterdam, 3015 GD The Netherlands; 2 Department of Medical Oncology, Netherlands Cancer Institute—Antoni van Leeuwenhoek, Amsterdam, 1066 CX The Netherlands; 3 Dutch AYA Care Network, Utrecht, 3511 DT The Netherlands; 4 Center of Expertise for Cancer Survivorship, Radboud University Medical Center, Nijmegen, 6525 GA the Netherlands; 5 Department of Medical Oncology, University Medical Center Groningen, University of Groningen, Groningen, 9713 GZ the Netherlands; 6 Department of Medical Oncology, Erasmus MC Cancer Center, Erasmus University Medical Center, Rotterdam, 3015 GD The Netherlands; 7 Department of Surgical Oncology, Erasmus MC Cancer Institute, Erasmus University Medical Center, Rotterdam, 3015 GD the Netherlands

**Keywords:** Adolescent and young adult cancer survivors, Late effects, Hospitalization, Long-term outcomes

## Abstract

**Purpose:**

To examine the long-term risk of hospitalization for medical conditions in adolescent and young adult (AYA) cancer survivors compared to a cancer-free AYA cohort.

**Methods:**

Retrospective cohort study of 42,653 ≥ 2-year cancer survivors diagnosed at ages 18–39 (1993–2016) in the Netherlands Cancer Registry, and 169,342 matched cancer-free controls (1:4 by age, sex and municipality). Hospitalizations for relevant medical conditions were identified through national hospital admission data. Survivors and controls were followed through December 2021. Hospitalization incidence rate ratios (IRRs) and cumulative incidences were calculated, with IRRs stratified by tumour type and therapy (chemotherapy, radiotherapy).

**Results:**

AYA cancer survivors had a significantly increased IRR of hospitalization for any medical condition (adjusted IRR 1.73; 95% CI 1.64 to 1.82), as well as for most individual conditions. The cumulative incidence of hospitalization for any medical condition was 25.4% at 25 years post-index, with most individual conditions below 5%. Most tumour subgroups had significantly increased IRRs compared to controls, with the highest IRRs observed for Hodgkin lymphoma and leukaemia. In general, elevated IRRs were more pronounced among survivors who received chemotherapy or radiotherapy.

**Conclusions:**

AYA cancer survivors have an increased risk of hospitalization for most medical conditions compared to AYAs without a cancer history, though absolute long-term risk is low. As hospital admissions reflect only part of the clinical and societal burden, the findings may underestimate the total impact of late effects.

**Implications for Cancer Survivors:**

AYA cancer survivors may benefit from long-term, risk-based survivorship care focused on early detection and management of late effects, especially after chemotherapy and radiotherapy.

**Supplementary Information:**

The online version contains supplementary material available at https://doi.org/10.1007/s11764-026-01996-5.

## Introduction

The incidence of cancer among adolescents and young adults (AYAs) has been rising in recent years [1] with approximately 1.3 million cases globally in 2022, and the incidence is projected to increase further [2]. Advances in screening, diagnosis and treatment have led to a decrease in both cancer-related and overall mortality, with approximately 85% of AYA cancer patients surviving at least five years after diagnosis [3, 4]. Despite these encouraging survival rates, this growing population faces ongoing risks of long-term and late effects and psychological challenges from cancer and its treatments [5, 6].

Chronic health conditions, such as cardiovascular disease and osteoporosis, are important potential long-term risks for AYA cancer survivors [7, 8]. These medical conditions can significantly impact their quality of life and may increase the risk of late mortality [9, 10]. However, research on chronic health risks in AYA cancer survivors remains limited, as AYAs have traditionally been grouped with either children (< 18) or older adults (≥ 40) in both clinical care and research [11, 12]. AYAs represent a distinct cancer subgroup, characterized by unique epidemiology, tumour biology and life impact, which necessitates specialized clinical care and research approaches [13–16]. Therefore, findings from studies on childhood cancer or older adult survivors cannot be directly extrapolated to AYA cancer survivors, highlighting the need for AYA-specific research on long-term risks.

Most large-scale studies of AYA cancer survivors have focused primarily on mortality and second malignancy risk, with less attention given to chronic conditions [3, 17–19]. Moreover, previous studies that examined health outcomes beyond mortality and malignancy have often focused on specific diseases often seen in the AYA age such as Hodgkin’s disease or testicular cancer, or been restricted to region-specific registries, lacking nationwide coverage and limiting external validity [7, 20–22]. Overall, there remains a lack of robust large cohort studies that examine long-term risk of medical conditions in AYA cancer survivors [5, 19]. 

In the Netherlands, all cancer cases have been registered in the Netherlands Cancer Registry (NCR) since 1989, providing comprehensive data on cancer incidence, as well as detailed clinical information on tumour and treatment characteristics [23]. By combining this nationwide registry with hospital data, hospitalizations for medical conditions can be studied longitudinally. Therefore, we conducted a cohort study linking data from the NCR and Dutch Hospital Data (DHD) to examine the long-term risk of hospitalization for medical conditions in both AYA cancer survivors and AYAs without cancer. While not all medical conditions arising after cancer treatment will typically require hospitalization, identifying the most prevalent conditions that do is essential for establishing an evidence base for optimizing long-term surveillance strategies among AYA cancer survivors.

## Methods

### Study setting and study population

For this retrospective cohort study, data from the NCR, non-public data from Statistics Netherlands and data from the DHD were linked at the individual level. AYA cancer survivors were identified through the NCR, which has registered all histopathologically confirmed incident cancers in the Netherlands from 1989 onward [23]. In the Netherlands, the AYA age range is defined as 18–39 years based on the Dutch healthcare system, which differs from the U.S. definition of 15–39 years. Individuals were eligible for inclusion in the AYA cancer survivor cohort if diagnosed with a primary invasive cancer (i.e. malignant tumour, excluding benign and in situ tumours) at ages 18–39 years between 1993 and 2016, with the start year chosen based on the availability of hospital data from 1995 and follow-up beginning 2 years after diagnosis. AYA cancer survivors were required to survive ≥ 2 years post-diagnosis, and the start of follow-up (index date) was set to two years after diagnosis, as active treatment is typically completed within this timeframe. This criterion ensured the study focused on long-term outcomes rather than early complications related to cancer treatment. Additionally, eligible survivors had to reside in the Netherlands from 2 years after diagnosis until the end of follow-up (December 2021) or until death. Individuals with low-grade chondrosarcomas, basal cell carcinoma, myelodysplastic syndromes, Langerhans cell tumours, borderline ovarian tumours and carcinomas of the appendix were excluded due to incomplete registration of these tumour types in the NCR and the debate regarding the malignancy of some of these tumours.

A control group of cancer-free AYAs was identified through data linkage of the NCR with the Personal Records Database of Statistics Netherlands, containing information on all residents of the Netherlands. Only individuals who resided in the Netherlands from 1995 to the end of 2021, or until death, and were cancer-free at AYA age during the entire study period were eligible for inclusion in the control group. Controls were matched 4:1 to cases by birth year, sex and municipality, and were assigned the same index date as their matched case. A lower matching ratio was accepted if less than 4 controls were available. AYA cancer survivors for whom no controls were available were excluded. Matching by municipality ensured the selection of cancer-free controls from the same healthcare region as the cases, guaranteeing equal access to hospitals as the AYA cancer survivor cohort. All study participants were followed from index date to death, or the end of 2021, whichever came first.

This study was approved and the requirement of informed consent was waived by the Institutional Review Board of the Netherlands Cancer Institute.

### Study variables

The primary outcome was hospitalization for selected medical conditions after the index date. Medical conditions of interest were known late effects of cancer treatment, as previously described by Chao et al. and Hayek et al. [7, 8]. These conditions included cardiovascular disease (ischaemic heart disease, cardiomyopathy/heart failure and stroke), diabetes, hyperlipidaemia, hypertension, thyroid disease, neurosensory conditions (hearing loss, vision loss, cataract and glaucoma), respiratory disease (asthma, COPD, emphysema and pulmonary fibrosis), musculoskeletal disease (fracture/avascular necrosis and osteoporosis), chronic liver disease or renal failure (Supplement 1).

Hospitalizations were retrieved from the DHD, which includes hospital admissions for the entire Dutch population, covering all general and academic hospitals, as well as four specialized institutions (two cancer clinics, one orthopaedic clinic and one eye hospital) [24–26]. The data include both day and clinical admissions, and both were regarded as hospitalizations in the analyses. Consequently, diagnostic procedures that required a day admission were also classified as hospitalization. Diagnoses were coded using the International Classification of Diseases, Ninth Revision (1995–2012) and Tenth Revision (2013–2021). Only hospitalizations in which one of the listed conditions was recorded as the primary diagnosis were included; admissions where the condition appeared only as a secondary diagnosis were excluded. This approach prevents conditions that commonly co-occur (e.g. cardiovascular disease and hyperlipidemia) from contributing to multiple hospitalization rates and thereby inflating estimates. Consequently, each hospitalization contributes to the rate of only the condition that was the primary reason for admission. Premature ovarian failure and depression and anxiety disorders, although noted by Chao et al. and Hayek et al. [7, 8] were not examined due to the rarity of hospitalizations associated with these diagnoses.

All-cause mortality during follow-up was determined using the nationwide Personal Records Database with information on date of death for all inhabitants of the Netherlands since 1994 [27]. Tumour and cancer treatment characteristics, as well as information on subsequent malignancies (i.e. second or higher-order primary cancer diagnoses, excluding recurrences; until 2022), for the AYA cancer survivor cohort were obtained from the NCR. Tumour types were categorized according to the AYA-specific tumour classification, established by Barr et al. [28, 29]. Sociodemographic variables were provided by Statistics Netherlands, including age, sex, migration status [30], household income level and educational level, based on the highest obtained educational qualification [31]. These variables were measured at the date of diagnosis, except for income and educational level, which were determined in the last available complete year, either the year before death or the last year of the study (2021).

### Statistical analysis

Descriptive statistics were computed for all patient characteristics. Differences between the two groups were assessed using chi-square tests for categorical variables and the Mann–Whitney *U* test for continuous variables. Post hoc analyses were conducted to explore differences within individual categories of the categorical variables when overall group differences were statistically significant. Incidence rates (IRs) of hospitalization for any of the included medical conditions (total), as well as for groups of conditions (cardiovascular disease, neurosensory conditions, respiratory disease and musculoskeletal disease) and for each individual condition, were calculated per 1000 person-years at risk in both groups. Person-time at risk was defined from the index date until death or the end of the study period. Hospitalization days were censored from the risk time, meaning individuals were not considered at risk for subsequent hospitalizations (for the same or other conditions) during periods of inpatient care. Because hospitalization rates were calculated using person-time at risk, differential mortality during follow-up does not disproportionately influence the estimated rate ratios; therefore, no stratification by vital status was applied.

To compare incidence rates of hospitalization between groups, negative binomial regression was used to estimate the incidence rate ratios (IRRs), adjusted for age, sex and migration background. Absolute excess risks (AER) for hospitalization were also calculated per 1000 person-years, defined as the absolute difference in IRs between AYA cancer survivors and cancer-free AYA controls. Cumulative incidence functions for hospitalization were calculated and visually plotted using nonparametric methods accounting for competing risks (i.e. death or hospitalization due to other medical conditions). Group differences in cumulative incidence of hospitalization were assessed using Gray’s test [32].

Subgroup analyses of hospitalization IRRs for any medical condition were conducted between cancer-free controls and subgroups of the 10 most common tumour types, stratified by chemotherapy exposure (yes/no) and radiotherapy exposure (yes/no), using negative binomial regression, adjusted for age, sex and migration background. For breast carcinoma and testicular cancer, the analysis was sex-restricted and therefore did not include sex as a covariate. Chemotherapy and radiotherapy were analyzed as independent, non-mutually exclusive binary exposures; patients who received both treatments contributed to both exposure-specific subgroup analyses.

Cumulative incidence analyses with competing risks were conducted in R, while all other statistical analyses were performed using STATA software (version 16.1; Stata Corporation, College Station, TX, USA).

## Results

A total of 42,653 AYA cancer survivors were matched to 169,342 cancer-free AYA controls (Supplemental Fig. 1). The majority of individuals were female (61.4% of cancer survivors and 61.5% of controls; Table 1). The mean age at cancer diagnosis was 32.6 ± 5.3 years. The most common tumour types among survivors were breast carcinoma (22.1%), melanoma (20.3%) and testicular cancer (13.8%). At diagnosis, most tumours were classified as TNM stage 1 (40.5%). Cancer treatment varied widely, with the most common therapies including surgery (81.8%), systemic chemotherapy (38.6%) and radiotherapy (32.5%).

**Table 1:** Characteristics of the study population

	AYA cancer survivors (*n* = 42,653)	Cancer-free AYA cohort (*n* = 169,342)	*p*-value
Demographics			
Sex, female	26,184 (61.4%)	104,160 (61.5%)	0.65
Age (mean, SD)	32.6 ± 5.3	32.6 ± 5.3	0.99
Age by groups, years			1.00
18–24	4247 (10.0%)	16,844 (10.0%)	
25–30	8891 (20.8%)	35,324 (20.9%)	
31–39	29,515 (69.2%)	117,174 (69.2%)	
Migration background			< 0.001
No	37,362 (87.6%)	146,079 (86.3%)	< 0.001^d^
Moroccan	536 (1.3%)	2749 (1.6%)	< 0.001^d^
Turkish	677 (1.6%)	3580 (2.1%)	< 0.001^d^
Surinamese	551 (1.3%)	3080 (1.8%)	< 0.001^d^
Former Dutch Antillean	199 (0.5%)	795 (0.5%)	0.94^d^
Other non-western	588 (1.4%)	2479 (1.5%)	0.19^d^
Other western	2740 (6.4%)	10,580 (6.3%)	0.18^d^
Household income level^a^			< 0.001
Low	8863 (20.8%)	37,432 (22.1%)	< 0.001^d^
Mid	18,913 (44.3%)	84,051 (49.6%)	< 0.001^d^
High	11,079 (26.0%)	47,110 (27.8%)	< 0.001^d^
Unknown	3798 (8.9%)	749 (0.4%)	< 0.001^d^
Main breadwinner of household^a^			< 0.001
No	18,165 (42.6%)	76,481 (45.2%)	< 0.001^d^
Yes	20,690 (48.5%)	92,112 (54.4%)	< 0.001^d^
Unknown	3798 (8.9%)	749 (0.4%)	< 0.001^d^
Educational level^a^			< 0.001
Low	4530 (10.6%)	20,681 (12.2%)	< 0.001^d^
Mid	12,008 (28.2%)	51,456 (30.4%)	< 0.001^d^
High	12,081 (28.3%)	44,894 (26.5%)	< 0.001^d^
Unknown	14,034 (32.9%)	52,311 (30.9%)	< 0.001^d^
Follow-up, years (mean, SD)^b^	11.4 ± 6.9	13.3 ± 6.6	< 0.001
Range	0.0–27.0	0.0–27.0	
Alive end of study			< 0.001
Yes	35,229 (82.6%)	166,398 (98.3%)	
No	7424 (17.4%)	2944 (1.7%)	
Tumour characteristics			
Tumour type		NA	
1. Leukemias and related disorder	1466 (3.4%)		
2. Lymphomas	4325 (10.1%)		
2.1 Non-Hodgkin lymphoma	2416 (5.7%)		
2.2 Hodgkin lymphoma	2416 (5.7%)		
2.3 Myeloma	104 (0.2%)		
2.4 Cutaneous lymphomas	22 (0.1%)		
2.5 Other B-cell and T-cell lymphomas	37 (0.1%)		
2.6 Other lymphomas, specified and unspecified	16 (0.0%)		
3. CNS and other intracranial and intra spinal neoplasms	1693 (4.0%)		
4. Sarcomas	1387 (3.3%)		
5. Blood and lymphatic vessel tumours	136 (0.3%)		
6. Nerve sheath tumours	75 (0.2%)		
7. Gonadal and related tumours	6734 (15.8%)		
7.1 Testicular	5876 (13.8%)		
7.2 Ovary	625 (1.5%)		
7.3 Germ cell and trophoblastic, and other	233 (0.5%)		
8. Melanoma, malignant	8661 (20.3%)		
9. Carcinomas	18,061 (42.3%)		
9.1 Thyroid carcinoma	1565 (3.7%)		
9.2 Other carcinoma of head and neck	679 (1.6%)		
9.3 Carcinoma of gastrointestinal tract	1828 (4.3%)		
9.4 Carcinoma of lung, bronchus and trachea	384 (0.9%)		
9.5 Carcinoma of skin	487 (1.1%)		
9.6 Carcinoma of breast	9433 (22.1%)		
9.7 Carcinoma of genital sites, excluding ovary and testis	2923 (6.9%)		
9.8 Carcinoma of urinary tract	591 (1.4%)		
9.9 Other invasive carcinomas	171 (0.4%)		
10. Miscellaneous specified neoplasms	76 (0.2%)		
11. Unspecified malignant neoplasms except CNS	39 (0.1%)		
TNM stage		NA	
I	17,259 (40.5%)		
II	7999 (18.8%)		
III	3690 (8.7%)		
IV	1056 (2.5%)		
Not applicable or unknown	12,649 (29.7%)		
Year of diagnosis		NA	
1993–1997	5821 (13.7%)		
1998–2002	7929 (18.6%)		
2003–2007	9004 (21.1%)		
2008–2012	10,449 (24.5%)		
2013–2016	9450 (22.2%)		
**Treatment characteristics**			
Primary cancer treatment		NA	
Surgery	34,908 (81.8%)		
Systemic chemotherapy	16,477 (38.6%)		
Local chemo/immunotherapy	201 (0.5%)		
Radiotherapy	13,863 (32.5%)		
Combined systemic chemotherapy and radiotherapy	7950 (18.6%)		
Targeted therapy	2290 (5.4%)		
Systemic hormonal therapy	4083 (9.6%)		
Stem cell transplantation	644 (1.5%)		
Metastasis surgery	2190 (5.1%)		
Metastasis radiotherapy	235 (0.6%)		
Other	829 (1.9%)		
Unknown	66 (0.2%)		
**Subsequent malignancy**^**c**^		NA	
No	39,062 (91.6%)		
Yes	3591 (8.4%)		

*p*-values calculated with Mann–Whitney test (continuous) and chi-square test (categorical)

^a^Measured in the last full available year, i.e. in case of death the year before the date of death or 2021 if still alive by then. Income level and bread winner information were available from 2011 to 2021; the educational level was available from 1999 to 2021. In case of death before these dates, the variable is missing

^b^Index date (2 years after diagnosis) up to death or end of the study (end of 2021), controls receive the same index date as the matched case

^c^This includes second primary cancer diagnoses; recurrences of the primary cancer diagnoses were excluded

^d^Post hoc analysis using the chi-square test

The mean follow-up from index was 11.4 ± 6.9 years among AYA cancer survivors (13.4 years from diagnosis) and 13.3 years among the cancer-free AYA cohort. During follow-up, 1636 (3.8%) AYA cancer survivors developed a subsequent malignancy. At the end of the study, 82.6% of AYA cancer survivors were still alive, compared to 98.3% of AYA cancer-free controls (*p* < 0.001).

### Hospitalization

During follow-up, AYA cancer survivors had an IR of hospitalization for any medical condition of 12.3 per 1000 person-years at risk, compared to 8.1 in cancer-free controls, resulting in an AER of 4.1 and an adjusted IRR of 1.73 (95% CI 1.64 to 1.82; Table 2). The adjusted IRR of hospitalization for each individual medical condition was statistically significantly increased for almost all conditions, except for asthma and emphysema. The IRR of hospitalization was most elevated for osteoporosis (adjusted IRR 15.15 [7.99 to 28.71]), liver disease (adjusted IRR 7.38 [4.71 to 11.57]), renal failure (adjusted IRR 4.85 [3.19 to 7.39]) and heart failure (adjusted IRR 3.68 [2.96 to 4.57]). Despite these high IRRs, the absolute risks remained low, with corresponding cumulative incidences in AYA cancer survivors of 0.6%, 0.6%, 1.3% and 2.1%, respectively, at 25 years after index.

**Table 2:** Hospitalization rates for medical conditions in AYA cancer survivors and cancer-free AYA cohort

Hospitalization	AYA cancer survivor (*n* = 42,653)			Cancer-free AYA cohort (*n* = 169,342)					
	No. of events	IR^a^	Cumulative incidence through end of study (25 years after index)	No. of events	IR^a^	Cumulative incidence through end of study (25 years after index)	AER	Adjusted IRR^a^ (95% CI)	*p*-value
Any medical condition^b^	5938	12.3	25.4%	18,235	8.1	22.7%	4.1	1.73 [1.64 to 1.82]	< 0.001
Cardiovascular disease^c^	2296	4.7	12.1%	7585	3.4	10.7%	1.4	1.60 [1.48 to 1.72]	< 0.001
Ischemic heart disease	1,409	2.9	7.7%	5305	2.4	7.6%	0.5	1.34 [1.22 to 1.47]	< 0.001
Heart failure	460	0.9	2.1%	757	0.3	1.0%	0.6	3.68 [2.96 to 4.57]	< 0.001
Stroke	444	0.9	2.2%	1560	0.7	2.1%	0.2	1.41 [1.24 to 1.60]	< 0.001
Respiratory disease^d^	727	1.5	3.4%	2717	1.2	4.0%	0.3	1.50 [1.23 to 1.82]	< 0.001
Asthma	267	0.6	1.1%	1431	0.6	1.7%	−0.1	0.88 [0.65 to 1.20]	0.42
COPD	389	0.8	2.0%	1089	0.5	1.9%	0.3	2.14 [1.65 to 2.77]	< 0.001
Emphysema	15	0.0	0.0%	84	0.0	0.0%	0.0	0.96 [0.38 to 2.43]	0.93
Pulmonary fibrosis	62	0.1	0.2%	126	0.1	0.3%	0.1	3.25 [1.85 to 5.70]	< 0.001
Musculoskeletal disease^e^	1177	2.4	3.9%	2,693	1.2	2.9%	1.2	2.25 [2.06 to 2.46]	< 0.001
Fracture	1001	2.1	3.4%	2574	1.1	2.8%	0.9	1.94 [1.77 to 2.12]	< 0.001
Osteoporosis	182	0.4	0.6%	124	0.1	0.1%	0.3	15.15 [7.99 to 28.71]	< 0.001
Other diseases									
Diabetes	556	1.1	1.7%	2109	0.9	1.7%	0.2	1.25 [1.06 to 1.47]	< 0.01
Hyperlipidemia	57	0.1	0.2%	169	0.1	0.1%	0.0	1.66 [1.16 to 2.39[	< 0.01
Hypertension	303	0.6	1.0%	1085	0.5	1.0%	0.1	1.39 [1.18 to 1.64]	< 0.001
Thyroid disease	222	0.5	0.8%	665	0.3	0.7%	0.2	1.63 [1.33 to 1.98]	< 0.001
Neurosensory	186	0.4	0.9%	741	0.3	1.1%	0.1	1.25 [1.02 to 1.53]	< 0.05
Liver disease	178	0.4	0.6%	260	0.1	0.3%	0.3	7.38 [4.71 to 11.57]	< 0.001
Renal failure	393	0.8	1.3%	542	0.2	0.5%	0.6	4.85 [3.19 to 7.39]	< 0.001

*IR*, incidence rate; *IRR*, incidence rate ratio; *AER*, absolute excess risk. Cumulative incidence calculated using nonparametric methods accounting for competing risk, i.e. death and for the separate medical conditions hospitalization for any other of the medical conditions. IRR estimated with multivariable negative binominal regression, adjusted for age, sex and migration background

^a^Per 1000 person years at risk

^b^Multiple diagnoses can be registered per hospitalization. Hospitalizations with multiple diagnoses are counted as 1 event in the number of total hospitalizations. For the separate morbidities, hospitalizations are calculated separately

^c^Ischemic heart disease, heart failure, stroke

^d^Asthma, COPD, emphysema, pulmonary fibrosis

^e^Fracture including avascular necrosis, osteoporosis

**Fig. 1:**
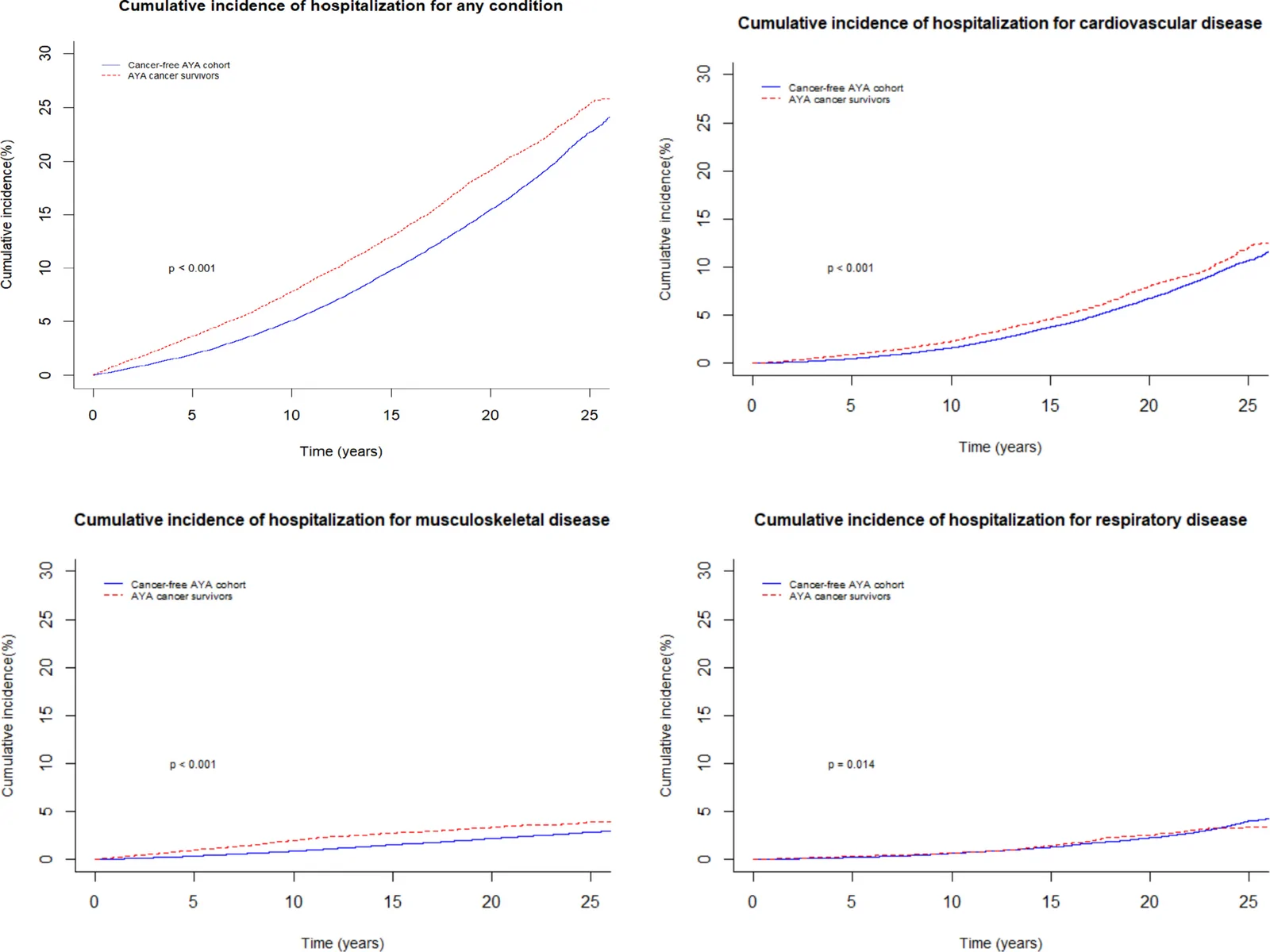
Cumulative incidences of hospitalization among AYA cancer survivors (*n* = 42,653) and cancer-free AYA controls (*n* = 169,342). Cumulative incidence calculated using nonparametric methods accounting for competing risk, i.e. death and for the separate medical conditions hospitalization for any other of the medical conditions

The cumulative incidence of hospitalization for any medical condition was 25.4% in AYA cancer survivors, with the cumulative incidence curve differing significantly from the cancer-free AYA cohort (Table 2, Fig. 1; *p* < 0.001). Among all groups of conditions and individual conditions, only cardiovascular disease and ischaemic heart disease reached a cumulative incidence above 5%, with a significantly different cumulative incidence curve for cardiovascular disease but not for ischaemic heart disease compared with controls (12.1% vs 10.7%, *p* < 0.001; 7.7% vs 7.6%, *p* = 0.06, respectively) (Table 2, Fig. 1 and Supplemental Fig. 2).

### Tumour subgroup analyses

**Fig. 2:**
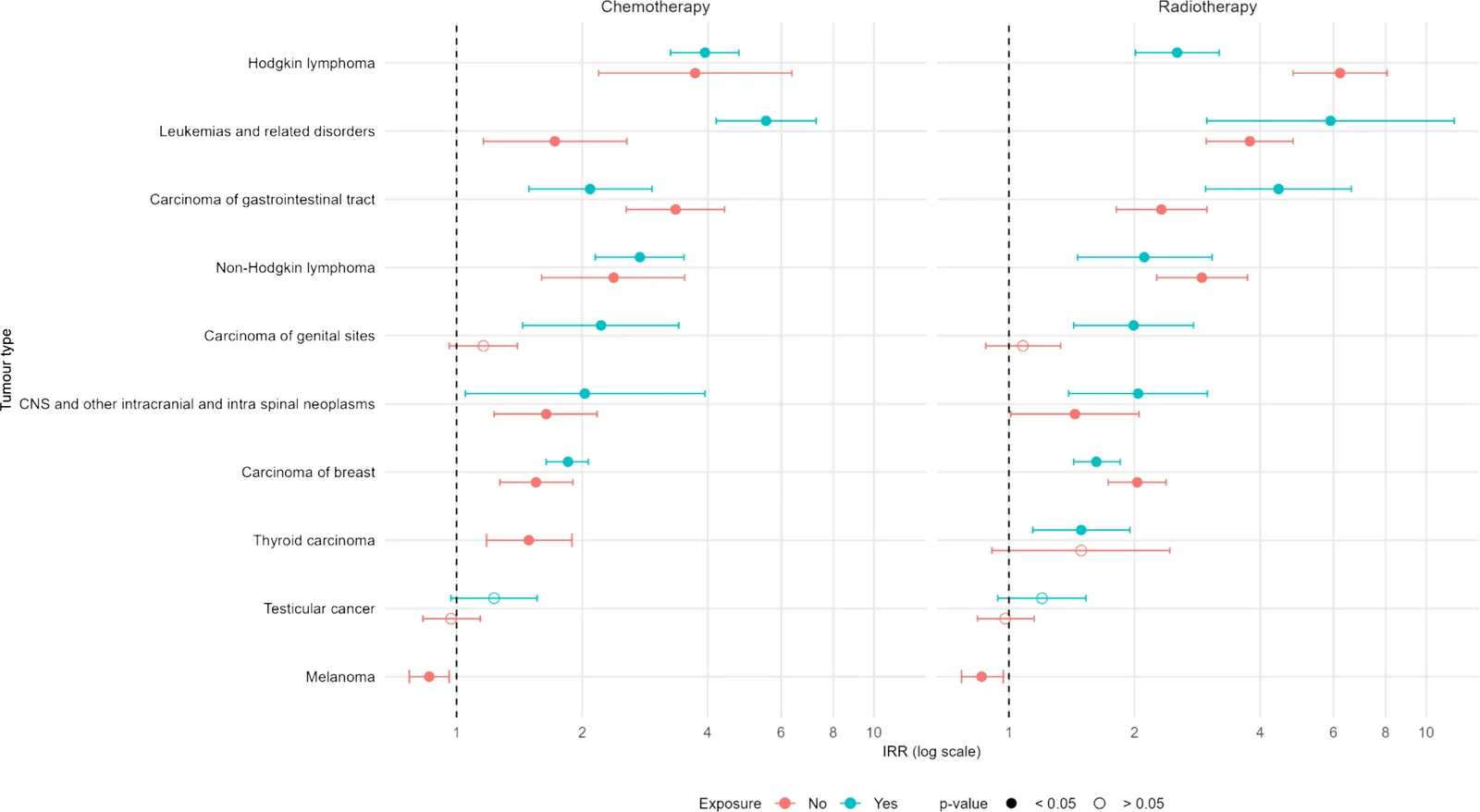
Increase contrast of list of cancer types. Incidence rate ratios of hospitalizations for any medical condition among AYA cancer survivors vs cancer-free AYA cohort, stratified by tumour type and therapy exposure. IRR estimated with multivariable negative binominal regression, adjusted for age, sex and migration background. Testicular cancer and breast carcinoma analyses were sex-restricted and not adjusted for sex. IRR per 1000 person years at risk. IRR not shown for those with melanoma who received chemotherapy or radiotherapy and those with thyroid carcinoma who received chemotherapy because of a low number of observations

Most tumour subgroups of AYA cancer survivors had a significantly increased adjusted IRR of hospitalization for any medical condition compared to cancer-free controls, regardless of chemotherapy and radiotherapy exposure (Fig. 2, Supplemental Table 2). Overall, survivors of Hodgkin lymphoma and leukaemia exhibited the highest significantly increased adjusted IRRs.

Within treatment strata, AYA survivors who received chemotherapy generally had higher adjusted IRRs of hospitalization than those who did not, relative to cancer-free controls (Fig. 2, Supplemental Table 2). This difference was particularly pronounced among sarcoma survivors, with an adjusted IRR of 2.27 for those receiving chemotherapy versus 1.28 for those who did not. Furthermore, among survivors of leukaemia, carcinoma of the gastrointestinal tract, genital site carcinoma and CNS tumours, those who received radiotherapy showed greater adjusted IRRs of hospitalization compared to those who did not receive radiotherapy. Among survivors of genital site carcinoma, only those who received chemotherapy or radiotherapy had a significantly increased adjusted IRR of hospitalization.

Those with testicular cancer did not demonstrate a significantly increased IRR of hospitalization compared to the cancer-free AYA age cohort, regardless of chemotherapy and radiotherapy exposure. Almost all survivors of melanoma did not receive chemotherapy or radiotherapy and had a significantly lower IRR of hospitalization compared to controls.

## Discussion

In this population-based study, we examined hospitalizations for medical conditions among AYA cancer survivors diagnosed between 1993 and 2016. Compared to a cancer-free AYA cohort, survivors had a significantly increased risk of hospitalization for most medical conditions during follow-up. However, absolute risks remained low for most conditions, with cumulative incidence estimates below 5% at 25 years post-diagnosis.

The medical conditions examined in this study were classified using the same ICD codes as those employed by Chao et al. and Hayek et al. [7, 8]. While our IRR of hospitalization for any medical condition (1.73) aligns with these earlier findings (1.47 and 1.60), our cumulative incidence estimates were notably lower. This likely reflects differences in how disease events were ascertained. Whereas prior studies incorporated procedural and medication codes in addition to diagnosis codes to identify chronic conditions [7, 8], our analysis was limited to diagnosis recorded during hospital admissions. This approach captures more severe health events requiring inpatient care and may offer a more specific estimate of the serious long-term health burden among AYA cancer survivors, which appears relatively low. Our findings are consistent with other studies that also relied solely on hospital admissions and reported similarly moderate risk increases in AYA cancer survivors across diagnostic groups [21, 33]. 

The relatively low hospitalization rates observed in this study likely reflect only part of the overall burden of late effects among AYA cancer survivors. Many chronic or less severe medical conditions are typically managed in outpatient settings and are thus underrepresented or absent in hospitalization data. This is illustrated by the low cumulative incidence rates observed for most individual medical conditions, which were generally below 5%. To contextualize these findings, a prior study reported notably higher cumulative incidences for second primary cancers, with 8.9% in males and 10.3% in females at 25 years post initial cancer diagnosis [34], suggesting that the clinical burden of subsequent malignancies exceeds that of hospitalizations for other conditions. To fully understand the complete health burden among cancer survivors, future research should incorporate outpatient diagnostic and treatment data to capture the broader spectrum of morbidity, including conditions managed during the early post-treatment phase in ambulatory care. This should also include psycho-oncological conditions, which were not captured in the current study, as somatic and psychological conditions often interact and together have a substantial impact on survivors’ quality of life [35]. 

An exception to the generally low hospitalization rates for most conditions was cardiovascular disease, which showed a cumulative incidence of 12.1% at 25 years post-diagnosis. This elevated risk may be attributable to cardiotoxic cancer treatments such as certain chemotherapies and radiation [36–38], along with modifiable factors such as physical inactivity and psychosocial stress following treatment [36, 39, 40]. In addition, the concept of accelerated aging caused by cancer therapies, including cellular damage, telomere shortening and chronic inflammation, may play an important role in the early development of cardiovascular disease [41–44]. In the control group, however, the cumulative incidence was also relatively high, likely due to the inclusion of both clinical and day admissions, combined with long-term follow-up into middle adulthood, when cardiovascular risk increases in the general population. This likely contributed to the relatively small difference between survivors and controls. The modest effect size (IRR 1.60) may additionally reflect underrepresentation in inpatient settings or cardiovascular prevention and management efforts among AYA cancer survivors.

Subgroup analyses revealed that survivors of Hodgkin lymphoma and leukaemia had the highest IRRs of hospitalization for any medical condition during follow-up, consistent with previous studies [8, 21, 33]. Although CNS tumour survivors are also reported to be at high risk for late-effect hospitalizations [8, 21, 33], they were not among the highest in our study, likely because a large proportion died during follow-up (57.6%; data not shown), potentially before late effects could develop.

Among leukaemia survivors, chemotherapy exposure had a marked impact, probably reflecting the intensive and prolonged regimens often administered to this group [45]. Across most other tumour groups, survivors who received chemotherapy also showed higher hospitalization IRRs compared to those who did not. Radiotherapy effects were less consistent, likely depending on treatment field and dose [46], but still contributed to an elevated rate of hospitalization in some tumour subgroups. These findings indicate that long-term morbidity is shaped by both tumour type and treatment, often through their combined effects. Survivorship care should therefore account for both factors rather than tumour type alone. For instance, survivors of genital site carcinoma only showed an increased hospitalization IRR compared to controls when treated with chemotherapy or radiotherapy, whereas those who did not receive these treatments did not have elevated hospitalization rates, highlighting the importance of treatment-focused follow-up. In addition, melanoma survivors generally did not receive chemotherapy or radiotherapy and showed a decreased hospitalization IRR compared to controls, which may reflect the absence of treatment-related late effects and potentially more favorable health behaviors following diagnosis. Future research should consider cumulative dose, time since diagnosis and sociodemographic factors to better identify survivors most vulnerable to late effects.

Several limitations should be considered. First, relying on hospital admissions to assess medical conditions likely underestimates the late effects of cancer and its treatment, particularly for conditions that primarily manifest in outpatient settings (e.g. diabetes, hypertension) yet significantly impact lifespan and quality of life. To better understand the full (clinical) burden in AYA cancer survivors, future research should incorporate data from outpatient care. Second, it is possible that some hospitalizations were missed due to misclassification, whereby medical conditions were registered with cancer codes as the primary diagnosis rather than the medical condition itself. Third, the DHD includes data from all hospitals and some specialized institutions but does not cover all clinics, which may have led to an underestimation of incidence rates and cumulative incidences. Fourth, because the NCR started in 1989, cancer cases diagnosed before that year could not be identified, so a small number of AYA cancer survivors may be misclassified as cancer-free, though this is expected to be minimal and unlikely to bias results. Moreover, since the NCR data used only includes cancers diagnosed during AYA age, controls with childhood or post-39 cancers overlapping the follow-up period could not be identified or excluded.

Despite these limitations, this study is among the largest to examine known late-effect medical conditions leading to hospitalization in AYA cancer survivors. The large population size, long follow-up and inclusion of a broad range of tumour types strengthen the robustness and generalizability of our findings.

## Conclusion

AYA cancer survivors have a significantly increased risk of hospitalization for medical conditions compared to AYAs without cancer. However, the absolute risk remains low even 25 years after diagnosis. Hospitalization rates vary substantially by tumour type and treatment exposure, with generally those who received chemotherapy and survivors of leukaemia and Hodgkin lymphoma experiencing the highest risk. Since hospital admissions represent only a partial measure of the broader clinical and societal burden, including outpatient-managed conditions and impacts on quality of life, the total burden of late effects in this population is likely underestimated. Future research should investigate outpatient care needs in this population to better understand the full spectrum of medical condition and survivorship challenges.

## Supplementary Information

Below is the link to the electronic supplementary material.

ESM 1 (DOCX 114 KB)

## Data Availability

The data underlying this article cannot be shared due to legal and privacy restrictions associated with the use of routine healthcare data. Data were obtained from Dutch Hospital Data (DHD), the Netherlands Cancer Registry (NKR) and Statistics Netherlands (CBS). Access to these data can be requested from the respective data providers upon formal application and approval.
