# Supplementary material for: Hospitalization for medical conditions in adolescent and young adult cancer survivors: a nationwide cohort study using the Netherlands Cancer Registry
Source: J Cancer Surviv. 2026 Mar 7;20(Suppl 2):1649–60. doi: 10.1007/s11764-026-01996-5 (PMC13744467; doi:10.1007/s11764-026-01996-5)
Supplement: Supplementary file 1 [file 11764_2026_1996_MOESM1_ESM.docx]

# **Supplementary material**

| **Supplemental Table 1. Definitions of medical conditions** | | |
| --- | --- | --- |
| Medical conditions | Definition | |
|  | ICD-9 | ICD-10 |
| Cardiovascular disease | Having at least one of the following cardiovascular disease (Ischemia heart disease, cardiomyopathy/heart failure, stroke) | |
| Ischemic heart disease | 410; Acute myocardial infarction  411; Other acute and subacute forms of ischemic heart disease  413; Angina pectoris  414; Other forms of chronic ischemic heart disease  V45.81; Aortocoronary bypass status convert  V45.82; Percutaneous transluminal coronary angioplasty status | I20[.0189]; Angina pectoris  I21[.012349]; Acute myocardial infarction  I22[.0189]; Subsequent myocardial infarction  I23[.01234568]; Certain current complications following acute myocardial infarction  I24[.0189]; Other acute ischaemic heart diseases  I25[.012345689]; Chronic ischaemic heart disease  I70[.01289]; Atherosclerosis  I71[.012345689]; Aortic aneurysm and dissection  I72[.012345689]; Other aneurysm and dissection  I73[.0189]; Other peripheral vascular diseases  I74[.01234589]; Arterial embolism and thrombosis  I77[.012345689]; Other disorders of arteries and arterioles  I78[.0189]; Diseases of capillaries  I79[.0128]; Disorders of arteries, arterioles and capillaries in diseases classified elsewhere  I11.0; Hypertensive heart disease with (congestive) heart failure  I13.0; Hypertensive heart and renal disease with (congestive) heart failure  I13.2; Hypertensive heart and renal disease with both (congestive) heart failure and renal failure  Z95.1; presence of aortacoronary bypass graft |
| Cardiomyopathy/heart failure | 425; Cardiomyopathy  428; Heart failure  V42.1; Heart replaced by transplant | I42[.0123456789]; cardiomyopathy  I50.0; Congestive heart failure  I50.1; Left ventricular failure  I50.9; Heart failure, unspecified  Z94.1; heart transplant status |
| Stroke | 430; Subarachnoid hemorrhage  431; Intracerebral hemorrhage  432; Other and unspecified intracranial hemorrhage  433; Occlusion and stenosis of precerebral arteries  434; Occlusion of cerebral arteries  435; Transient cerebral ischemia | I60[.0123456789]; Subarachnoid haemorrhage  I61[.012345689]; Intracerebral haemorrhage  I62[.019]; Other nontraumatic intracranial haemorrhage  I63[.012345689]; Cerebral infarction  I64; Stroke, not specified as haemorrhage or infarction  I65[.012389]; Occlusion and stenosis of precerebral arteries, not resulting in cerebral infarction  I66[.0123489]; Occlusion and stenosis of cerebral arteries, not resulting in cerebral infarction  I67[0.123456789]; Other cerebrovascular diseases  I68[.0128]; Cerebrovascular disorders in diseases classified elsewhere  I69[.012348]; Sequelae of cerebrovascular disease |
| Diabetes | 250.xx; diabetes mellitus  357.2; polyneuropathy in diabetes  362.0; diabetic retinopathy  366.41; cataract associated with other disorder  648.0; diabetes mellitus complicating pregnancy childbirth or the puerperium | E10[.0123456789]; Type I diabetes mellitus  E11[.0123456789]; Type 2 diabetes mellitus  O24[.012349]; diabetes mellitus in pregnancy  E13.3; other specified diabetes mellitus with ophthalmic complications |
| Hyperlipidemia | 272.0; Pure hypercholesterolemia  272.1; Pure hyperglyceridemia  272.2; Mixed hyperlipidemia  272.3; Hyperchylomicronemia  272.4; Other and unspecified hyperlipidemia | E78.0; Pure hypercholesterolaemia  E78.1; Pure hyperglyceridaemia  E78.2; Mixed hyperlipidaemia  E78.3; Hyperchylomicronaemia  E78.4; Other hyperlipidaemia  E78.5; Hyperlipidaemia, unspecified |
| Hypertension | 401.X; Essential hypertension  402.X; Hypertensive heart disease  403.X; Hypertensive kidney disease  404.X; Hypertensive heart and kidney disease  405.X; Secondary hypertension | I10; Essential (primary) hypertension  I11.9; Hypertensive heart disease without (congestive) heart failure  I12.9; Hypertensive renal disease without renal failure  I13.0; Hypertensive heart and renal disease with (congestive) heart failure  I13.2; Hypertensive heart and renal disease with both (congestive) heart failure and renal failure  I13.1; Hypertensive heart and renal disease with renal failure  I15.0; Renovascular hypertension  I15.1; Hypertension secondary to other renal disorders  I15.2; Hypertension secondary to endocrine disorders  I15.8; Other secondary hypertension  I15.9; Secondary hypertension, unspecified |
| Thyroid disease | 240.xx; Simple and unspecified goiter  241.xx; Nontoxic nodular goiter  242.xx; Thyrotoxicosis with or without goiter  244.xx; Acquired hypothyroidism  245.xx; Thyroiditis  246.xx; Other disorders of thyroid | E04.1; Nontoxic single thyroid nodule  E04.2; Nontoxic multinodular goitre  E04.9; Nontoxic goitre, unspecified  E05.0; Thyrotoxicosis with diffuse goitre  E05.1; Thyrotoxicosis with toxic single thyroid nodule  E05.2; Thyrotoxicosis with toxic multinodular goitre  E05.3; Thyrotoxicosis from ectopic thyroid tissue  E05.4; Thyrotoxicosis factitia  E05.8; Other thyrotoxicosis  E05.9; Thyrotoxicosis, unspecified  E89.0; Postprocedural hypothyroidism  E03.2; Hypothyroidism due to medicaments and other exogenous substances  E03.4; Atrophy of thyroid (acquired)  E03.8; Other specified hypothyroidism  E03.9; Hypothyroidism, unspecified  E01.8; Other iodine-deficiency-related thyroid disorders and allied conditions  E06.0; Acute thyroiditis  E06.1; Subacute thyroiditis  E06.3; Autoimmune thyroiditis  E06.4; Drug-induced thyroiditis  E06.5; Other chronic thyroiditis  E06.9; Thyroiditis, unspecified  E07.8; Other specified disorders of thyroid  E07.9; Disorder of thyroid, unspecified |
| Neurosensory | Having any neurosensory condition (vision loss, hearing loss, cataract, and glaucoma surgery) | |
| Hearing loss | 389.xx; Hearing loss | H90[.012345678]; Conductive and sensorineural hearing loss |
| Vision loss | 369.xx; Blindness and low vision  12.1; Iridotomy and simple iridectomy  12.64; Trabeculectomy ab externo  12.7; Other procedures for relief of elevated intraocular pressure | H44.5; Degenerated conditions of globe  H53.6; Night blindness  H54[.01234569]; Visual impairment including blindness (binocular or monocular)  H47.6; Disorders of visual cortex |
| Cataract | 'V45.61' | H26.0; Infantile, juvenile and presenile cataract  H26.2; Complicated cataract  H26.8; Other specified cataract  H26.9; Cataract, unspecified |
| Glaucoma | '12.1; 12.64; 12.7; Z98.89' | H40.0; Glaucoma suspect  H40.1; Primary open-angle glaucoma  H40.2; Primary angle-closure glaucoma  H40.8; Other glaucoma  H40.9; Glaucoma, unspecified  H42.0; Glaucoma in endocrine, nutritional and metabolic diseases  H42.8; Glaucoma in other diseases classified elsewhere |
| Respiratory disease | Any chronic condition from the respiratory conditions (asthma, COPD, emphysema, pulmonary fibrosis) | |
| Asthma | 493.xx; asthma | J45[.0189]; Asthma |
| COPD | 496.xx; Chronic airway obstruction, not elsewhere classified | J44[.0189]; Other chronic obstructive pulmonary disease |
| Emphysema | 492.xx; emphysema | J43[.01289]; Emphysema |
| Pulmonary fibrosis | 515.xx; Post inflammatory pulmonary fibrosis | J84.1; Other interstitial pulmonary diseases with fibrosis  J84.8; interstitial pulmonary diseases  J84.9; Interstitial pulmonary disease, unspecified |
| Musculoskeletal disease | Having at least one of the musculoskeletal diseases (Fracture/Avascular necrosis and joint replacement, osteoporosis) | |
| Fracture /Avascular necrosis | Fracture  820.xx; fracture of neck of femur  821.xx; fracture of other and unspecified parts of femur  822.xx; fracture of patella  823.xx; fracture of tibia and fibula  824.xx; fracture of ankle  825.xx; fracture of one or more tarsal and metatarsal bones  826.xx; fracture of one or more phalanges of foot  827.xx; other, multiple, and ill-defined fractures of lower limb  828.xx; multiple fractures involving both lower limbs, lower with upper limb and lower limb(s) with rib(s) and sternum  829.xx; fracture of unspecified bones  733.1; pathologic fracture  733.8; malunion and nonunion of fracture  Avascular necrosis  733.4; Aseptic necrosis of bone  733.40; Aseptic necrosis of bone, site unspecified  733.41; Aseptic necrosis of head of humerus  733.42; Aseptic necrosis of head and neck of femur  733.43; Aseptic necrosis of medial femoral condyle  733.44; Aseptic necrosis of talus  733.45; Aseptic necrosis of bone, jaw  733.49; Aseptic necrosis of bone, other | S72[.01234789]; fracture of femur  S82[.0123456789]; fracture of lower leg, including ankle  S92[.01234579]; fracture of foot, except ankle  M84.4; pathological fracture, not elsewhere classified  Avascular necrosis:  M87.0; Idiopathic aseptic necrosis of bone  M80.0; postmenopausal osteoporosis with pathological fracture  M87.8; Other osteonecrosis  M87.9; osteonecrosis, unspecified |
| Osteoporosis | 733.0; Osteoporosis  733.00; Osteoporosis, unspecified  733.01; Senile osteoporosis  733.02; Idiopathic osteoporosis  733.03; Disuse osteoporosis  733.09; Other osteoporosis | M81[.012345689]; osteoporosis without pathological fracture |
| Chronic liver disease and cirrhosis | 571.xx; Chronic liver disease and cirrhosis | K74[.0123456]; Fibrosis and cirrhosis of liver  K76.0; Fatty (change of) liver, not elsewhere classified  K76.9; Liver disease, unspecified  K76.8; Other specified diseases of liver |
| Chronic renal failure/CKD | 585.xx; chronic renal failure | N18[.123459]; Chronic kidney disease |

Individuals with cancer diagnosis at AYA age in NCR
n= 90,255

Eligible for inclusion
n = 42,659

Excluded total n = 47,596

Reason

1. Low-grade chondrosarcomas, myelodysplastic syndromes, Langerhans cell tumours, borderline ovarian tumours, carcinomas of the appendix

(n = 1,921)

2. Cancer diagnosis before 1993 (n = 2,084); of whom 400 develop a secondary cancer between 1993-2016)

3. Cancer diagnosis after 2016 (n = 17,460)

4. Passed away within two years after diagnosis (n = 8,438)

5. Not a resident in the Netherlands during the entire study period (1995-2022) (and did not pass away) (n = 7,064)

6. Not linkable with hospital data (n = 10,369)

Individuals (birth years 1954-1998) without cancer and resident in the Netherlands during the entire study period (1995-2021) and linkable with all other data sources
n= 4,423,306

Eligible for inclusion
n = 4,423,306

1:4 matching by birth year, sex, and municipality

Cases
n= 42,653

Controls
n= 169,342

**Supplemental Figure 1. Flowchart of study population selection**

**Supplemental Figure 2. Cumulative incidence curves of hospitalization**

| **Supplemental Table 2. IRR of hospitalization for any medical condition by tumour group and therapy exposure (reference = non-cancer AYA cohort)** | | | | | |
| --- | --- | --- | --- | --- | --- |
| Tumour type | Exposure | | n | Adjusted IRR (95% CI) | p-value |
| Leukemias and related disorders | Chemotherapy | Yes | 967 | 5.52 [4.19 – 7.28] | <0.001 |
|  |  | No | 499 | 1.72 [1.16 – 2.56] | 0.007 |
|  | Radiotherapy | Yes | 151 | 5.90 [2.98 – 11.68] | <0.001 |
|  |  | No | 1315 | 3.78 [2.97 – 4.80] | <0.001 |
| Non-Hodgkin Lymphoma | Chemotherapy | Yes | 1245 | 2.75 [2.15 – 3.51] | <0.001 |
|  |  | No | 485 | 2.38 [1.60 – 3.52] | <0.001 |
|  | Radiotherapy | Yes | 530 | 2.11 [1.46 – 3.07] | <0.001 |
|  |  | No | 1200 | 2.90 [2.26 – 3.73] | <0.001 |
| Hodgkin Lymphoma | Chemotherapy | Yes | 2205 | 3.94 [3.26 – 4.75] | <0.001 |
|  |  | No | 211 | 3.73 [2.19 – 6.36] | <0.001 |
|  | Radiotherapy | Yes | 1336 | 2.53 [2.01 – 3.19] | <0.001 |
|  |  | No | 1080 | 6.22 [4.80 – 8.06] | <0.001 |
| CNS and other intracranial and intra spinal neoplasms | Chemotherapy | Yes | 307 | 2.03 [1.05 – 3.94] | 0.037 |
|  |  | No | 1386 | 1.64 [ 1.23 – 2.17] | 0.001 |
|  | Radiotherapy | Yes | 843 | 2.04 [1.39 – 2.99] | <0.001 |
|  |  | No | 850 | 1.44 [1.01 – 2.05] | 0.047 |
| Testicular cancer | Chemotherapy | Yes | 1743 | 1.23 [0.97 – 1.56] | 0.088 |
|  |  | No | 4133 | 0.97 [0.83 – 1.14] | 0.742 |
|  | Radiotherapy | Yes | 1312 | 1.20 [0.94 – 1.53] | 0.140 |
|  |  | No | 4564 | 0.98 [0.84 – 1.15] | 0.842 |
| Melanoma | Chemotherapy | No | 8646 | 0.86 [0.77 – 0.96] | 0.009 |
|  | Radiotherapy | No | 8570 | 0.86 [0.77 – 0.97] | 0.010 |
| Thyroid carcinoma | Chemotherapy | No | 1564 | 1.49 [1.18 – 1.89] | 0.001 |
|  | Radiotherapy | Yes | 1231 | 1.49 [1.14 – 1.95] | 0.004 |
|  |  | No | 334 | 1.49 [0.91 – 2.43] | 0.113 |
| Carcinoma of gastrointestinal tract | Chemotherapy | Yes | 814 | 2.09 [1.49 – 2.94] | <0.001 |
|  |  | No | 1014 | 3.35 [2.55 – 4.39] | <0.001 |
|  | Radiotherapy | Yes | 449 | 4.43 [2.96 – 6.62] | <0.001 |
|  |  | No | 1379 | 2.32 [1.81 – 2.98] | <0.001 |
| Carcinoma of breast | Chemotherapy | Yes | 7415 | 1.85 [1.64 – 2.07] | <0.001 |
|  |  | No | 1996 | 1.55 [1.27 – 1.90] | <0.001 |
|  | Radiotherapy | Yes | 6031 | 1.62 [1.43 – 1.85] | <0.001 |
|  |  | No | 3380 | 2.03 [1.73 – 2.38] | <0.001 |
| Carcinoma of genital sites | Chemotherapy | Yes | 482 | 2.22 [1.44 – 3.41] | <0.001 |
|  |  | No | 2441 | 1.16 [0.96 – 1.40] | 0.124 |
|  | Radiotherapy | Yes | 759 | 1.99 [1.43 – 2.77] | <0.001 |
|  |  | No | 2164 | 1.08 [0.88 – 1.33] | 0.441 |
| IRR estimated with multivariable negative binominal regression, adjusted for age, sex, and migration background. Testicular cancer and breast carcinoma analyses were sex-restricted and not adjusted for sex. IRR per 1000 person years at risk. IRR not shown for those with melanoma who received chemotherapy or radiotherapy and those with thyroid carcinoma who received chemotherapy because of a low number of observations. | | | | | |
